# MiniCD4 Microbicide Prevents HIV Infection of Human Mucosal Explants and Vaginal Transmission of SHIV_162P3_ in Cynomolgus Macaques

**DOI:** 10.1371/journal.ppat.1003071

**Published:** 2012-12-06

**Authors:** Nathalie Dereuddre-Bosquet, Laurence Morellato-Castillo, Joachim Brouwers, Patrick Augustijns, Kawthar Bouchemal, Gilles Ponchel, Oscar H. P. Ramos, Carolina Herrera, Martha Stefanidou, Robin Shattock, Leo Heyndrickx, Guido Vanham, Pascal Kessler, Roger Le Grand, Loïc Martin

**Affiliations:** 1 CEA, Division of Immuno-Virology, iMETI, Fontenay-aux Roses, France; 2 Paris-Sud University, UMRE01, Orsay, France; 3 CEA, iBiTecS, Service d'Ingénierie Moléculaire des Protéines, Gif sur Yvette, France; 4 Katholieke Universiteit Leuven, Leuven, Belgium; 5 Paris-Sud University, Faculty of Pharmacy, Chatenay-Malabry, France; 6 Section of Infectious Diseases, Faculty of Medicine, St Mary's Campus, Imperial College, London, United Kingdom; 7 Department of Pediatrics and Microbiology-Immunology, Albert Einstein College of Medicine, Bronx, New York, United States of America; 8 Institute of Tropical Medicine and University of Antwerp, Antwerp, Belgium; Emory University, United States of America

## Abstract

In complement to an effective vaccine, development of potent anti-HIV microbicides remains an important priority. We have previously shown that the miniCD4 M48U1, a functional mimetic of sCD4 presented on a 27 amino-acid stable scaffold, inhibits a broad range of HIV-1 isolates at sub-nanomolar concentrations in cellular models. Here, we report that M48U1 inhibits efficiently HIV-1_Ba-L_ in human mucosal explants of cervical and colorectal tissues. *In vivo* efficacy of M48U1 was evaluated in nonhuman primate (NHP) model of mucosal challenge with SHIV_162P3_ after assessing pharmacokinetics and pharmacodynamics of a miniCD4 gel formulation in sexually matured female cynomolgus macaques. Among 12 females, half were treated with hydroxyethylcellulose-based gel (control), the other half received the same gel containing 3 mg/g of M48U1, one hour before vaginal route challenge with 10 AID_50_ of SHIV_162P3_. All control animals were infected with a peak plasma viral load of 10^5^–10^6^ viral RNA (vRNA) copies per mL. In animals treated with miniCD4, 5 out of 6 were fully protected from acquisition of infection, as assessed by qRT-PCR for vRNA detection in plasma, qPCR for viral DNA detection in PBMC and lymph node cells. The only infected animal in this group had a delayed peak of viremia of one week. These results demonstrate that M48U1 miniCD4 acts *in vivo* as a potent entry inhibitor, which may be considered in microbicide developments.

## Author Summary

This report describes the protective effect of a CD4 peptide mimetic against HIV infection on human mucosal explants and further on, when used in a microbicide gel, against a SHIV challenge in cynomolgus macaques. Evidence is given that our “miniprotein engineering” strategy, which corresponds to miniaturizing a binding protein by transferring its binding site onto a small scaffold protein, followed by an extensive optimization of this miniprotein led to an active peptide with promising pre-clinical antiretroviral activity in the case of the gp120/CD4 interaction. Five out of six animals, pre-treated with a 0.3% miniCD4 gel were fully protected from SHIV_162P3_ vaginal challenge. On the whole, we demonstrated that such a small CD4 mimetic peptide could represent a powerful preventive agent against sexual HIV transmission, validating miniaturized protein interface design up to the discovery of potential new drugs.

## Introduction

In complement to an effective vaccine against HIV transmission, development of potent anti-HIV microbicides remains important strategies to consider for HIV prevention [1]–[2]. The CAPRISA-004 clinical trial of 1% tenofovir gel provides the first encouraging results from a randomized phase IIb efficacy trial with a 39% reduced risk of HIV acquisition among HIV-uninfected woman [3]. In this study tenofovir gel was applied in a pericoital fashion both before and after intercourse. While encouraging, the observed levels of protection were less than optimal reaching only 50% in those reporting >80% adherence to the dosing strategy. More recently trial of a once-daily dosing regimen with tenofovir gel (VOICE) failed to demonstrate any detectable efficacy in at risk women. These studies underline the need to develop additional microbicide candidates with complementary or synergistic activity. Indeed, the combination of different antiretroviral candidates acting on different steps of the viral cycle and possessing non-overlapping resistance profiles might be a key to increasing the levels of protection observed in the CAPRISA-004 trial. However, only few microbicide candidates, in early clinical development, have been tested for efficacy against vaginal or rectal SIV/SHIV challenge in macaques, providing proof of concept for accelerated clinical development.

The use of effective therapeutic drugs, such as tenofovir and more recently dapivirine tested in phase III trial, targeting post-entry events in the viral replication cycle (reverse transcription, integration and viral maturation) remains attractive [3], however the impact of their dual use in prevention and therapy on the potential induction of resistance evolution remains controversial. In contrast drugs that target the initial steps of viral attachment to CD4 and one of two co-receptors (CCR5 or CXCR4) triggering viral fusion with susceptible targets cells (entry inhibitors) are not widely used in therapy. Some CCR5-inhibitors have demonstrated efficacy against vaginal challenge with simian-human immunodeficiency virus (SHIV) in macaque models [4]–[6]. While active against monotropic R5 virus exclusively utilizing the CCR5 co-receptor, these compounds would provide no protection against viruses either able to utilize both the CXCR4 and CCR5 co-receptors (dual tropic, R5X4 virus) or against monotropic X4 viruses exclusively utilizing CXCR4 for viral entry. However, compounds active against CD4 binding would likely be active against all three phenotypes of virus: R5, X4 and R5/X4. Few drugs target CD4 expression, one notable exception being CADA, yet to be evaluated in non human primate (NHP) studies [7]. Furthermore the consequence of interference with this key immunoregulatory protein on the immune system has not been fully characterized. In contrast the use of compounds directly targeting the CD4 binding site in the viral envelope protein (gp120) is less likely to have any immunomodulatory effects, providing a promising alternative strategy. Previous studies demonstrated that a small molecule inhibitor of gp120-CD4 interaction (BMS-378806) could protect NHP from vaginal challenge with SHIV_162P3_ [8]. However, the breadth of activity for this compound against the diversity of natural viral isolates was too narrow to warrant further development as a microbicide candidate.

Here we describe the evaluation of a miniCD4 mimetic, M48U1 presented on a stable 27 amino acid scaffold. Its close structural mimicry of CD4 endows it with broad activity across a wider range of pseudotyped virus expressing envelope of HIV-1 isolates [9]. We report the activity of M48U1 against HIV infection of human cervical and colorectal human tissue explants. Furthermore we present NHP studies to access both the pharmacokinetics and efficacy of M48U1 against vaginal challenge with SHIV_162P3_.

## Results/Discussion

Small peptide mimetics of CD4, called miniCD4s, have been developed by our group for a number of years through a process of binding site transfer and further extensive optimization of a scorpion toxin scaffold [10]–[11]. Structural studies confirmed the close binding similarity between CD4 and these compounds [12]–[13]. Among the most recent generation of miniCD4s, M48U1, which no longer contains any residue common with macaque or human CD4, was identified as possessing sub-nanomolar affinity for gp120 with potent antiviral activity against HIV-1 subtype B and C pseudoviruses with ED_50_ values ranging from 0.07 to 2 nM and 0.4 to 37 nM, respectively [9].

**Figure 1:**
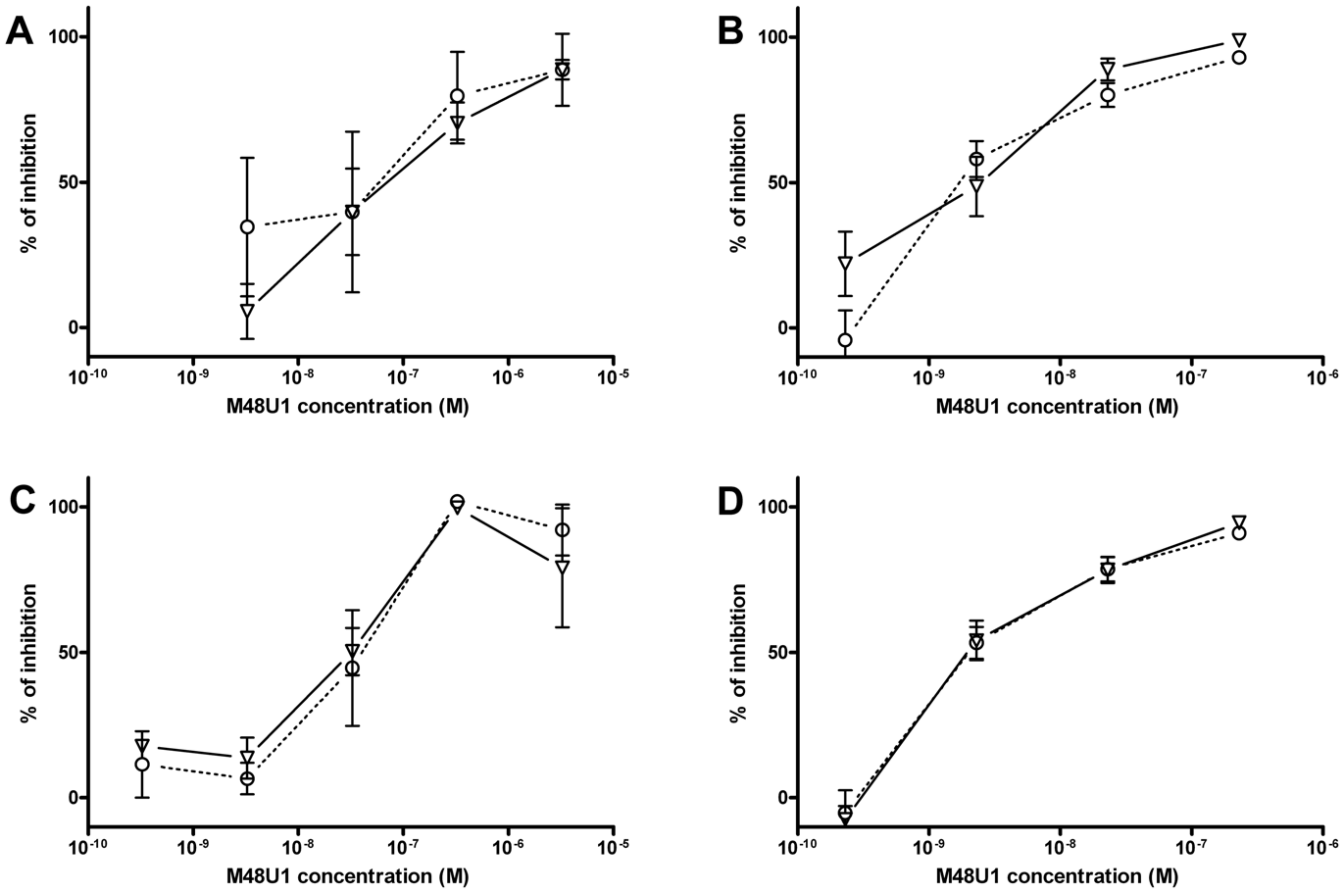
Inhibition of HIV-1_Ba-L_ infection by M48U1 in genital tissue explant models. Inhibition of HIV-1_Ba-L_ infection of cervical (**A**) and colorectal (**B**) tissue explants pre-treated with M48U1. (**C**) Inhibition of HIV-1_Ba-L_ trans-infection of PM-1 T cells co-cultured with cells migrating out of cervical tissue explants treated with M48U1. (**D**) Inhibition of HIV-1_Ba-L_ infection of colorectal tissue explants treated with M48U1 coming from dilution of the formulation at 0.3% in the gel used for in vivo challenge, comprising 1.5% hydroxyethylcellulose (HEC), 0.1% sorbic acid and 2.5% glycerol kept at 4°C during more than one year. Viral replication was assessed by measuring p24 production in supernatants at days 10 (circle, dotted line) and 15 (triangle, solid line). The percentage of inhibition was normalized relative to the p24 values obtained for explants not exposed to HIV-1_Ba-L_ (0% infectivity) and for explants exposed to HIV-1_Ba-L_ in the absence of M48U1 (100% infectivity). Data points correspond to mean values and error bars represent the standard error of the mean (SEM) from three independent experiments performed in triplicates.

**Figure 2:**
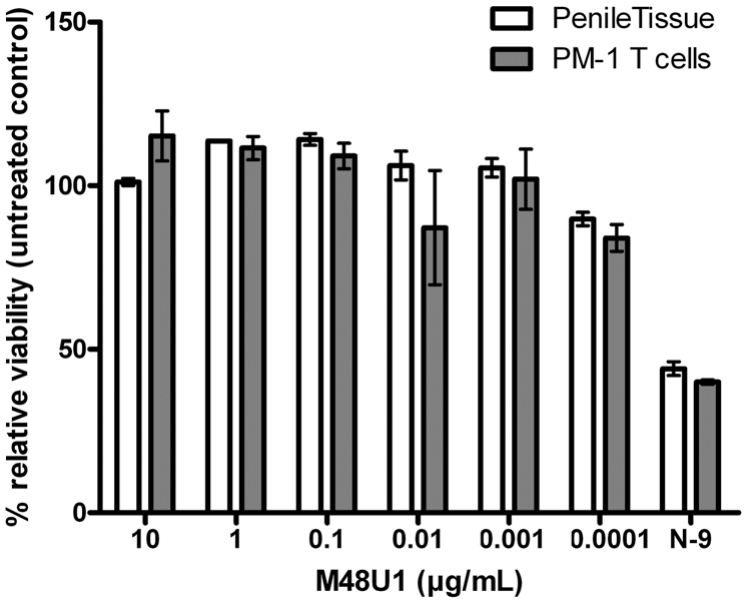
Viability assay to evaluate the cytotoxicity of M48U1 on mucosal tissue explants and PM-1 T cells. Penile tissue explants (blank bars) and PM-1 cells (grey bars) were exposed to 10-fold dilutions of M48U1 for 24 h at 37°C. After removing the peptide, the tissue explants and the cells were incubated with MTT for 2 h. Then the viability was determined by measuring the formazan release. Nonoxynol-9 microbicide (500 ng/mL) was employed as cytotoxic reference, while the untreated control was assumed as 100% of viability. Data points correspond to mean values and error bars represent the standard error of the mean (SEM) from two independent experiments performed in triplicates.

These positive results prompted further assessment of the activity of M48U1 in genital and colorectal tissue explants models. Nonpolarized cervical and colorectal explant tissues were pre-treated with M48U1 for 1 h at 37°C followed by exposure to HIV-1_Ba-L_ for 2 h in the continued presence of compound. M48U1 inhibited mucosal explant infection in the low nanomolar range with IC_50_ value of 42 nM (9–193 nM) and 4 nM (1–28 nM) for cervical tissue (Figure 1A) and in colorectal tissue (Figure 1B), respectively. Furthermore, M48U1 effectively inhibited the dissemination of HIV-1_Ba-L_ to co-cultured T cells (PM-1) by migratory cells (dendritic cells and CD4 T cells) that emigrate out of cervical explants during the first 24 h of culture [14] (Figure 1C) with an IC_50_ of about 42 nM (11–157 nM). A small diminution in response was seen at the highest concentration of drug tested. Presumably this effect is due to the variability of the model or it could also reflect some loss in solubility of M48U1 at neutral pH. In contrast, the miniCD4 is very soluble at pH 4.5 (up to 30 mg/mL), which is the pH of the HEC- gel formulation (see Materials and Methods) used for *in vivo* challenge. M48U1 formulated in the HEC-gel was thus tested in colorectal tissue and the HIV-1_Ba-L_ infection inhibition was as efficient as unformulated peptide (Figure 1D), displaying very tight error bars. Interestingly, M48U1 retained its anti-HIV activity in this gel even when kept for several months at 4°C. These data demonstrated that M48U1 miniCD4 was active against both direct mucosal tissue infection and dissemination of infection by migratory dendritic cells in *ex vivo* models. As some microbicides were shown to have poor biocompatibility and to induce inflammatory responses, we first evaluated the cytotoxicity of M48U1 using an enhanced colorimetric MTT assay. In co-cultures of monocyte-derived dendritic cells (MO-DC) and allogeneic CD4+ T cells, cultures of ME-180 endocervical cells [9], here, in penile tissue and in a T-cell line (PM-1), M48U1 showed no apparent cytotoxicity at the highest concentrations tested (Figure 2), suggesting good biocompatibility for the miniCD4. This was in contrast to the significant toxicity when the same tissue or cells were treated with nonoxynol-9 (Figure 2). Prior to macaque challenge studies, the activity of M48U1 was determined in TZM-bl cells against the simian-human chimeric virus (SHIV_162P3_) or pseudotyped SHIV_162P3_, with IC_50_ values of 25 nM and 9 nM, respectively (Table 1). These results were consistent with the nanomolar activity of M48U1 previously reported against various HIV-1 isolates [9].

**Table 1:** Inhibition activity of M48U1 in TZM-bl exposed to HIV-1_SF162_, SHIV_162P3_ or pseudovirus (PV) containing HIV-1_SF162_ or SHIV_162P3_ envelope.

Virus/pseudovirus	IC_50_ (nM)
HIV-1_SF162_	0.95
SHIV_162P3_	25
	Ratio SHIV/SF = 26
PV HIV-1_SF162_	0.85
PV SHIV_162P3_	9.36
	Ratio SHIV/SF = 11

**Figure 3:**
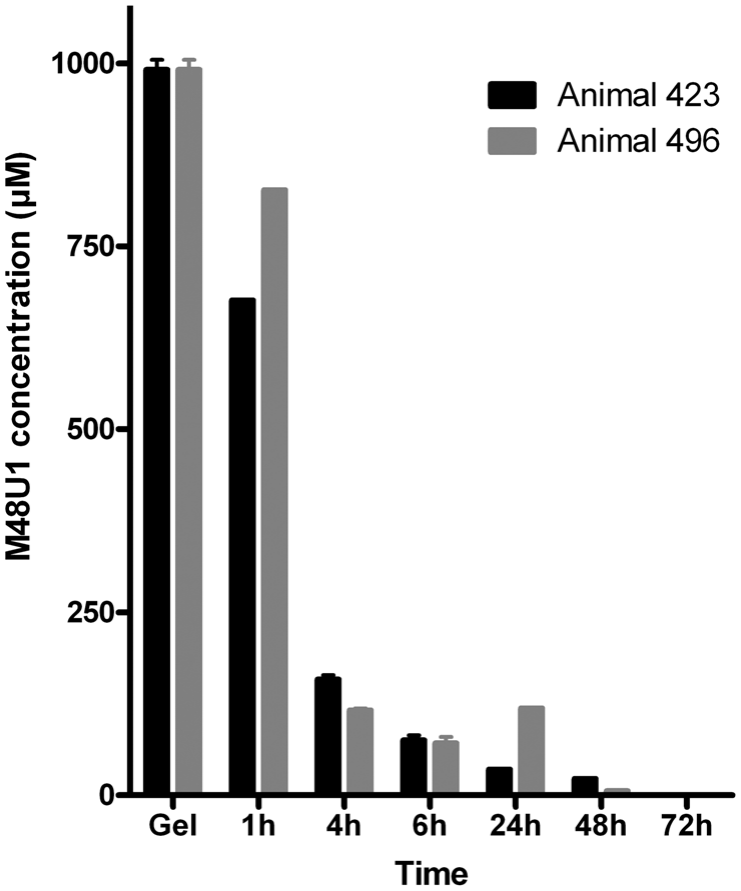
Pharmacokinetic studies in non-infected macaques. Mean concentration of M48U1 miniCD4 measured in the vaginal fluid of two cynomolgus macaques following vaginal administration of a single 2 g sample of 1.5% hydroxyethylcellulose (HEC) gel containing M48U1 at 3 mg/g (about 1 mM). The vaginal fluids were sampled before and 1 h, 4 h, 6 h, 24 h, 48 h and 72 h after gel application and analyzed by MS spectra with an internal reference for M48U1 quantification. The bars represent the standard error of the mean (SEM) of the three assay replicates.

Based on these encouraging data, macaque studies were initiated to assess the effectiveness of M48U1 against vaginal challenge with SHIV_162P3_. To determine optimal dose and appropriate time of viral challenge, formulation and pharmacokinetic studies were performed in uninfected animals. Previous reports indicated that inhibitor concentrations >1000 fold above the *in vitro* IC_50/90_ are required to protect macaques from *in vivo* mucosal challenge [4], [8]. Therefore, M48U1 was formulated at 0.3% (*i.e.* 3 mg/g of gel – corresponding to about 1 mM) in a gel comprising 1.5% hydroxyethylcellulose (HEC), 0.1% sorbic acid and 2.5% glycerol, at pH 4.6. One hour after M48U1-HEC gel application, peptide concentration in vaginal fluid was still about 75% of its concentration in the gel and this value dropped to 10% after 4 hours (Figure 3).

For SHIV challenge studies two groups of 6 naïve and sexually mature female cynomolgus macaques (*Macaca fascicularis*) were treated with medroxyprogesterone acetate (Depo-Provera, Pfizer, 30 mg) 40 days in advance. Animals received 2 g of HEC gel with (M48U1 group) or without (placebo group) M48U1 miniCD4. The gel was atraumatically instilled into the vaginal vault one hour before challenge. Intravaginal challenge was carried out with a 10-fold animal infectious dose 50% (AID_50_), corresponding to 2,500 tissue culture infectious dose 50% (TCID_50_) of SHIV_162P3_ (obtained from the NIH AIDS Research and Reference Reagent Program [15]), inoculated in 50% human seminal plasma [16].

**Figure 4:**
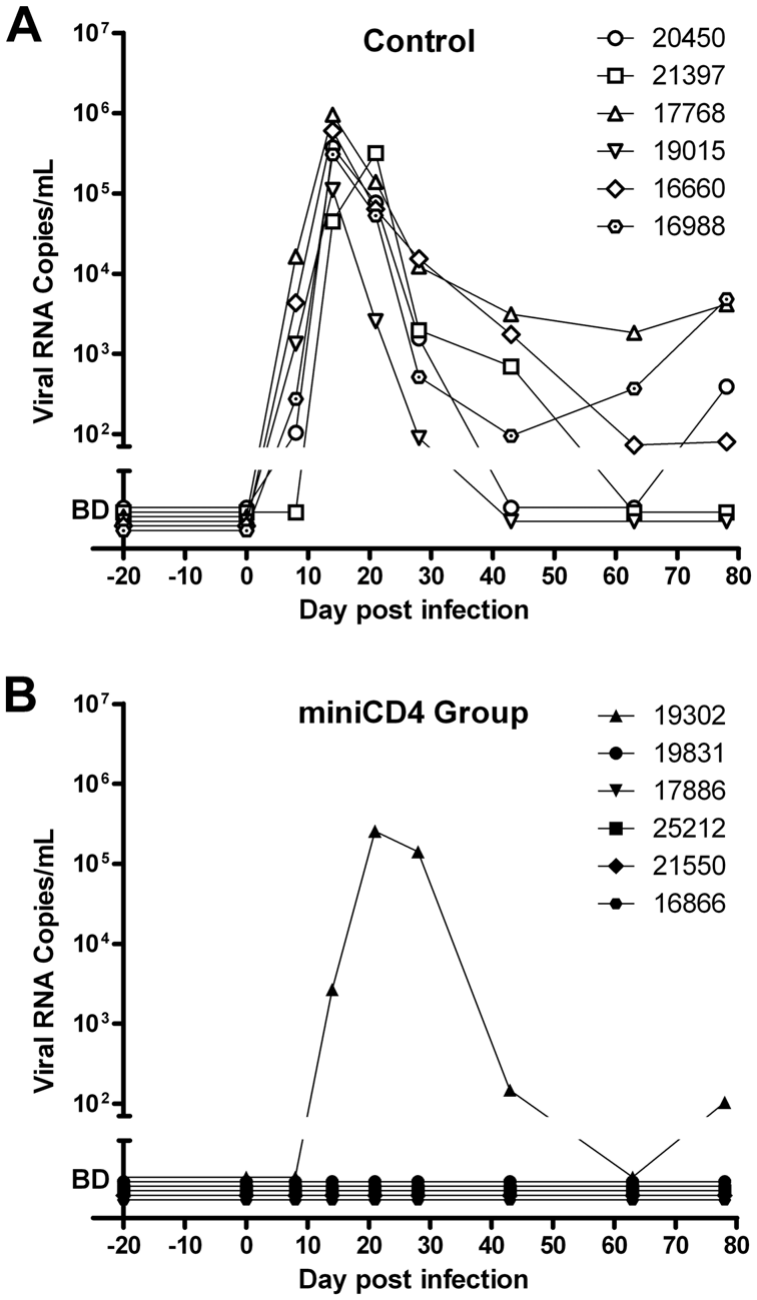
Protection of macaques against vaginal SHIV challenge by pretreatment with M48U1 gel. Viral loads in placebo (**A**) and M48U1-gel (**B**) treated macaques. One hour after gel application, animals were challenged with 10 AID_50_ of SHIV_162P3_. Macaques were monitored regularly for plasma viremia for 78 days. The area below the ordinate axis break represents values below the 60 RNA copies/mL detection limit (BD – below detection).

As expected, after this stringent ‘high dose’ challenge of progesterone-treated females, all the control animals became infected as evidenced by detection of virus in plasma, followed by seroconversion. In contrast, five out of six animals, treated with 0.3% M48U1 were fully protected resulting in a significant difference compared to control animals (p = 0.0152; Fisher exact test). For these five animals (Figure 4), plasma viral loads remained below detection limit (60 copies of vRNA/mL) throughout the duration of the study, they did not seroconvert and the SHIV DNA copy numbers in lymph nodes also remained below the limit of detection (10 copies/million cells). The plasma of the single animal (# 19302) infected in the M48U1 gel group was fully evaluated for possible miniCD4-resistant viruses. Plasma was collected 21 days after challenge, and the extracted virus genes were sequenced and pseudotyped. All the breakthrough viruses tested were fully susceptible to M48U1 in TZM-bl assay. Some mutations were observed (notably the Q507R mutation) but none of them were shown to be responsible for any form of resistance. We don't find any reported resistance mutations [17] in such breakthrough viruses. In addition, protected animals did not differ from control animals in terms of MHC haplotypes associated with control of viremia [18]–[20] (Supplemental Figure S1). Mucosal treatments could potentially induce local inflammatory response which may interfere with infection and microbicide effect. Quantities of cytokines and chemokines in vaginal fluids of macaques 25 h after treatment with placebo- or M48U1-gel were not significantly different (Supplemental Figure S2), except lower G-CSF (60%) in M48U1- *vs* placebo-treated animals. However, it is possible that imperfect gel delivery in the infected animal or the 0.3% dosing of M48U1 were not sufficient to achieve 100% protection. Further dose/response studies are required to eliminate this last possibility.

In conclusion, we have demonstrated that the CD4 mimetic M48U1 miniprotein can efficiently protect macaques from SHIV challenge, indicating that this small peptide, acting as a fusion entry inhibitor, could represent a new preventive agent against sexual transmission of HIV-1 when formulated as a microbicide. Thanks to its stably folded scaffold, this peptide possesses high stability and resistance to temperature and protease degradation. Contrary to other entry inhibitors, miniCD4 targets the virus and not a human receptor. Moreover, it is not used in current HIV therapy. This might provide a significant advantage as it may prevent the spread of viruses that become resistant to current treatments. Our study provided evidence that the molecules, which interact with the CD4-binding site of HIV envelope, are highly potent at blocking HIV entry and represent valuable microbicide candidates. Finally, these results show that “our miniprotein engineering strategy”, which corresponds to miniaturizing a protein by transferring its binding site onto a small scaffold followed by optimization, could represent an effective method to develop new binding-inhibitory drugs with modified physicochemical and pharmacokinetic properties.

## Materials and Methods

### Ethics statement

Adult cynomolgus macaques (*Macaca fascicularis*) were imported from Mauritius and housed in the facilities of the “Commissariat à l'Energie Atomique et aux Energies Alternatives” (CEA, Fontenay-aux-Roses, France). Non-human primates (NHP, which includes *M. fascicularis*) are used at the CEA in accordance with French national regulation and under national veterinary inspectors (CEA Permit Number A 92-032-02). The CEA is in compliance with Standards for Human Care and Use of Laboratory of the Office for Laboratory Animal Welfare (OLAW, USA) under OLAW Assurance number #A5826-01. All experimental procedures were also conducted accordingly to European guidelines for animal care (European directive 86/609, “Journal Officiel des Communautés Européennes”, L358, December 18, 1986). The use of NHP at CEA is also in accordance with recommendation with the newly published European Directive (2010/63, recommendation N°9). No suffering was specifically associated with vaginal treatment of macaques. The animals were used under the supervision of the veterinarians in charge of the animal facility. This study was part of the European Microbicides Project (EMPRO, EU contract number LSHCT-2004-503558) which NHP studies were accredited by ethical committee “Comité Régional d'Ethique pour l'Expérimentation Animale Ile-De-France Sud” under statement number 04-001 of April 7^th^, 2004.

### M48U1 synthesis

The M48U1 peptide containing a *p*-(cyclohexylmethyloxy)phenylalanine residue at position 23 was synthesized at Pepscan Presto Inc. (Lelystad, The Netherlands) by solid phase peptide synthesis and purified after refolding by reverse-phase high performance liquid chromatography as described elsewhere [21].

### Viruses

SHIV_162P3_ virus was obtained through AIDS Research and Reference Reagent Program, NIAID, NIH from Drs Janet Harouse, Cecilia Cheng-Mayer, Ranajit Pal and the DAIDS, NIAID [15]. HIV-1_Ba-L_, provided by this Program was passaged through activated PBMC for 11 days.

### Model explant study

Cervical tissue was obtained from patients undergoing planned therapeutic hysterectomy at St. George's Hospital in London, United Kingdom. Surgically resected specimens of colorectal tissue were collected at St. George's Hospital, London, United Kingdom. Penile tissue (glans) was obtained from individuals undergoing gender reassignment surgery at Charing Cross Hospital. All tissues were collected after signed informed consent was received from all patients and under protocols approved by the Local Research Ethics Committee (National Research Ethic Service NHS). On arrival in the laboratory, resected tissue was cut into 2–3 mm^3^ explants comprising both epithelial and stromal tissue or muscularis mucosae, depending on the tissue, as described previously [14], [22].

Cervical explants were cultured in RPMI 1640 medium supplemented with 2 mM L-glutamine, 10% fetal calf serum (FCS), and antibiotics (100 U/mL of penicillin, 100 µg/mL of streptomycin) Cervical explants were pre-incubated with ten-fold dilutions of M48U1 for 1 h. Tissue was then exposed to HIV-1_Ba-L_ (at 10^4^ TCID_50_/mL equivalent to an MOI of 1) for 2 h and then washed 4 times with PBS to remove compound and/or virus. Explants were then transferred to a fresh tissue culture plate [14]. Following overnight incubation, tissue explants were moved to a fresh tissue culture plate and migratory cells left in the original plate were washed twice with PBS and co-cultured with 4×10^4^ PM-1 cells/well without compound in 96-well plates for trans-infection assays. Tissue explants and cellular co-cultures were cultured for 15 days in the absence of compound.

Colorectal explants were maintained with DMEM containing 10% FCS, 2 mM L-glutamine and antibiotics (100 U/mL of penicillin, 100 µg/mL of streptomycin, 80 µg/mL of gentamicin). Colorectal explants were incubated with ten-fold dilutions of M48U1 for 1 h before exposure to HIV-1_Ba-L_ (at 10^3^ TCID_50_/mL equivalent to an MOI of 1). Explants were then washed 4 times with PBS to remove unbound compound and/or virus. Tissue explants were then transferred onto gelfoam rafts (Welbeck Pharmaceuticals, UK) and cultured for 15 days as previously described [22] in the absence of compound.

For all tissue explant models and migratory/PM-1 cell co-cultures approximately 50% of the supernatants of explants and cellular co-cultures were harvested every 2 to 3 days following re-fed with fresh media in the presence or absence of compound. The extent of virus replication was determined by measuring the p24 antigen concentration in supernatants (HIV-1 p24 ELISA, AIDS Vaccine Program, National Cancer Institute, Frederick, MA) [22]. All culture and incubations were carried out at 37°C in an atmosphere containing 5% CO_2_.

### Viability assay on tissue explants and PM-1 T cells

Penile tissues and PM-1 T cells were incubated in complete RPMI medium supplemented with ten-fold dilutions of M48U1 for 24 h at 37°C. Nonoxynol-9 microbicide (tested at 500 ng/mL) was employed as cytotoxic reference. The compounds were removed and the tissue explants and cells were incubated for 2 h in medium containing 500 µg/mL of 3-(4,5-dimethylthiazol-2-yl)-2,5-diphenyltetrazolium bromide (MTT). For tissue, after releasing the formazan in ethanol overnight, cytotoxicity was determined by measuring the optical density of the purple formazan product at 570–690 nm using Synergy HT multi-detection microplate reader. For PM-1 cells, the absorption of the formazan product was determined after lysis of the cells.

### Evaluation of sensitivity of HIV-1_SF162_ and SHIV_162P3_ to M48U1 in the TZM-bl assay

Primary HIV-1_SF162_ or SHIV_162P3_ were briefly amplified in PHA-IL2 activated human PBMC. Corresponding Env expressing constructs were prepared by DNA amplification of the complete Env gene from PBMC co-cultures and subsequent cloning into pSV7d or pcDNA4/TO expression vector (Invitrogen BV, Groningen, The Netherlands). Sequencing of the pseudovirus construct and phylogenetic analysis of the complete gp160 confirmed the relation between the pseudovirus and the associated original virus.

Pseudoviruses were generated in a 24-well plate by co-transfection of 2×10^5^ human embryonic kidney (HEK) 293T cells (obtained from ATCC) with pNL4-3. LucR-E- (400 ng, NIH AIDS Research and Reference Reagent Program) and HIV-1_SF162_ or SHIV_162P3_ Env expressing plasmid (1 µg) using the Calcium phosphate method (ProFection Mammalian Transfection Systems, Promega Benelux BV, Leiden, The Netherlands). After 24 h, the culture medium (Dulbecco's Minimum Essential Medium - DMEM) was replaced with medium containing 1 mM sodium butyrate and incubated for another day. Two days post-transfection, pseudoviruses were harvested and passed through Millex 0.45 µm filters. 10% fetal bovine serum (FBS) was added to filtered pseudoviruses, then the suspensions were aliquoted in 1 mL tubes and stored at −80°C until use.

Adherent CD4 and CCR5 expressing TZM-bl cell line with a luciferase reporter gene under HIV LTR control (NIH AIDS Research and Reference Reagent Program) [23] was cultured in DMEM containing 1% L-glutamine, 10% heat-inactivated FBS, and 50 µg/mL gentamicin.

Primary viruses and pseudoviruses were preliminarily titrated in TZM-bl cells and used at a concentration that resulted in 10^5^ relative light units (RLU). The inhibitory activity of M48U1 against either HIV-1_SF162_, SHIV_162P3_ or corresponding pseudoviruses was measured as follows: 50 µL of (pseudo)virus suspension and 50 µL of a serial dilution of M48U1 or 50 µL medium (negative control) were pre-incubated for 30 min. Next, 100 µL of TZM-bl cells (at 10^5^/mL) supplemented with 30 µg/mL DEAE dextran were added to each well and the 96-well plates were incubated for 48 h. Subsequently, 120 µL of supernatants were removed and 75 µL of Steadylite HTS (Perkin Elmer, Life Sciences, Zaventem, Belgium) were added. Next the luciferase activity (proportional to the amount of infectious virus particles) was measured using a TriStar LB941 luminometer (Berthold Technologies GmbH & Co.KG., Bad Wildbad, Germany) and expressed as relative light units (RLU). Finally, the inhibitory activity was calculated in GraphPad Prism 5.03 using non-linear regression (GraphPad Software, San Diego, CA, USA). All cultures and incubations were carried out at 37°C in 5% CO_2_ atmosphere.

### Gel formulation of M48U1

M48U1 was formulated at 3 mg/g in an aqueous vehicle containing sorbic acid (0.1%), glycerol (2.5%) and gelling polymer hydroxyethylcellulose (HEC, final concentration 1.5%). The pH of the gels was 4.6±0.1, within the range of the normal, premenopausal vaginal pH. The osmolality (292±3 mOsm/kg) fell within the range of physiological fluids, thereby avoiding safety issues with hyperosmolar microbicide gels. Placebo gels were prepared in the same way but did not contain any M48U1.

### Macaque Pharmacokinetic (PK) study

Two grams of the previously described gel containing M48U1 at 3 mg/g were injected, using a French catheter connected to ready-to-use syringe, into the vaginal vault of two naïve female cynomolgus macaques (*Macaca fascicularis*). Vaginal fluids were sampled using Weck-Cel surgical sponges before and 1 h, 4 h, 6 h, 24 h, 48 h and 72 h after gel application. Only one sampling was performed for each animal. Multiple samplings would increase the statistical analysis but would result in a decrease of the miniCD4 concentration in the vagina for the next samplings. The miniCD4 concentration of each sample was independently assessed 3 times and the resulting data was represented by mean and standard error of the mean (SEM). To determine these concentrations, MS spectra were registered with a 4800 MALDI-TOF/TOF mass spectrometer (Applied Biosystems, Foster City, USA) using an internal standard consisting of M48U3 (similar peptide with a slight difference of residue at position 23) [9].

### Macaque challenge study

Two groups of 6 naïve female cynomolgus macaques (*Macaca fascicularis*), treated with 30 mg medroxyprogesterone acetate (Depo-Provera, Pfizer) 40 days before the challenge, were atraumatically injected into the vaginal vault 1 h before challenge with 2 g of HEC gel with (M48U1 group) or without (placebo group) M48U1. Intravaginal challenge was carried out with 10 AID_50_ of SHIV_162P3_, corresponding to 2,500 TCID_50_, inoculated in 50% human seminal plasma [16].

Blood was collected in EDTA tubes at different time points, from 20 days before to 78 days after infection. Plasma viremia for each sample was evaluated using quantitative RT-PCR for measurement of viral RNA copy numbers. Detection limit of this method is 60 RNA copies/mL and quantification limit is 300 RNA copies/mL [24]. The SHIV DNA copy numbers in lymph nodes were also measured in animals with undetectable plasma viremia by quantitative PCR, using primers amplifying the gag regions of SHIV. Detection limit is 10 copies per million of cells [25].

Vaginal fluids were collected 24 h after viral challenge, *i.e.* 25 h after intravaginal treatment. Cytokines and chemokines (G-CSF, GM-CSF, IFN-γ, IL-1β, IL-1Ra, IL-2, IL-5, IL-6, IL-8, IL-10, IL-12/23(p40), IL-17, IL-18, MCP-1, MIP-1α, MIP-1β, TNF-α) were quantified using non-human primate Milliplex kit (Millipore) and a Bioplex2000 instrument (Biorad).

## Supporting Information

Figure S1 **MHC haplotypes of the Mauritian cynomolgus macaques included in the study.** MHC haplotypes were determined by microsatellite analysis as described elsewhere [26]. M1 to M6 haplotypes were identified in these macaques (M1, black; M2, red; M3, blue; M4, green; M5, yellow; M6, grey). White boxes indicate variant microsatellite allele sizes relative to the expected haplotype. These rare variants generally differ by the addition or loss of a single repeat unit.
(TIF)

Figure S2 **Cytokine and chemokine in macaque vaginal fluids after placebo- or M48U1 gel intravaginal administration.** Vaginal fluids were collected 25 h after intravaginal application of either placebo- or M48U1-gel, *i.e.* 24 h after SHIV_162P3_ challenge. Individual data and mean with standard error of the mean (SEM) are presented. Group comparison was performed using non parametric Mann-Whitney test. Elevated TNF-α concentrations (not significant) were observed in two M48U1-treated animals (#16866: 26 pg/mL and #19831: 174 pg/mL). Both animals were protected from SHIV acquisition, suggesting that such TNF-α levels were not associated with enhancement of infection.
(TIF)
