# Supplementary figures and images for: MiniCD4 Microbicide Prevents HIV Infection of Human Mucosal Explants and Vaginal Transmission of SHIV162P3 in Cynomolgus Macaques
Source: PLoS Pathog. 2012 Dec 6;8(12):e1003071. doi: 10.1371/journal.ppat.1003071 (PMC3516572; doi:10.1371/journal.ppat.1003071)

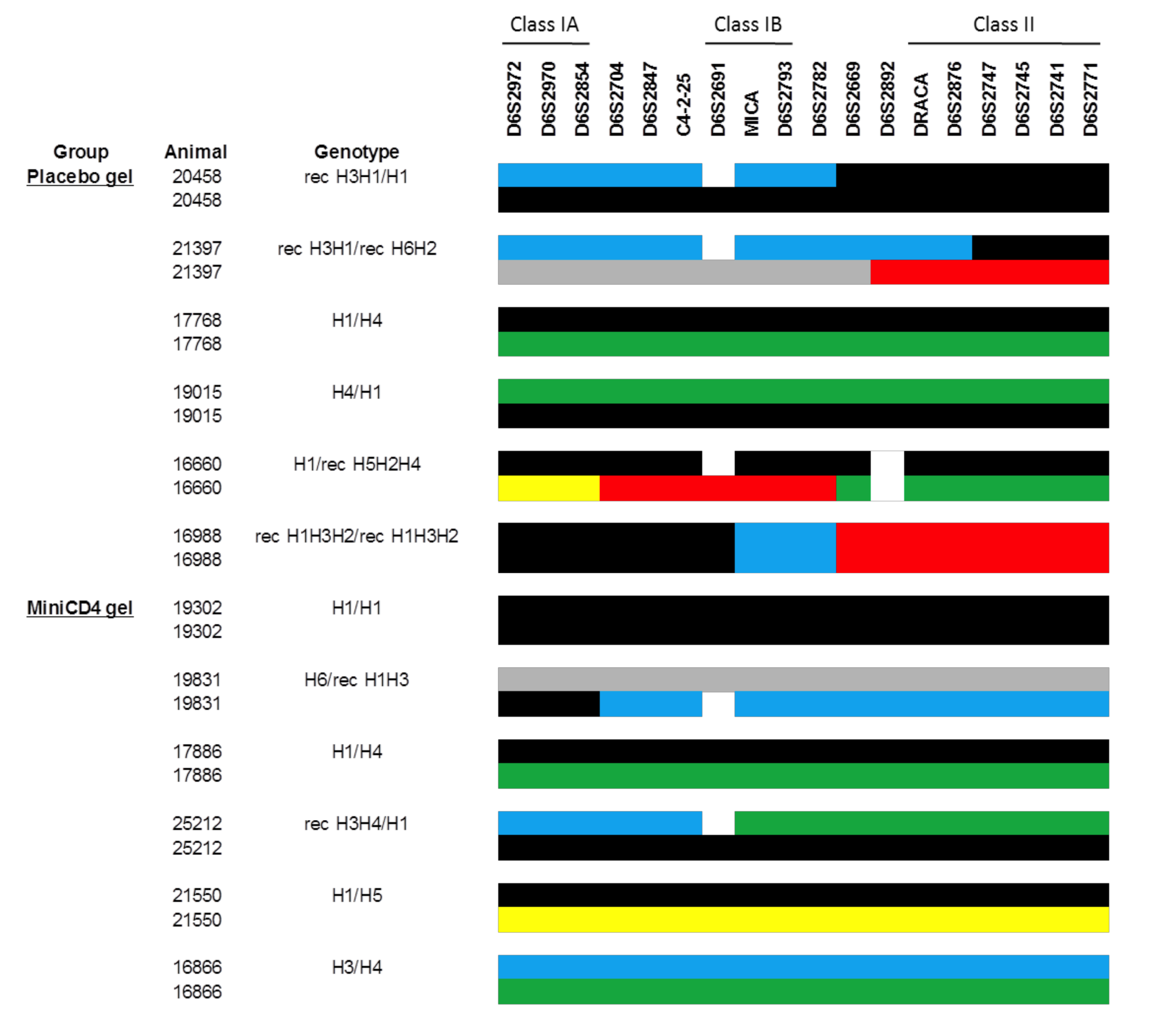

Supplement: Figure S1 — MHC haplotypes of the Mauritian cynomolgus macaques included in the study. MHC haplotypes were determined by microsatellite analysis as described elsewhere [26]. M1 to M6 haplotypes were identified in these macaques (M1, black; M2, red; M3, blue; M4, green; M5, yellow; M6, grey). White boxes indicate variant microsatellite allele sizes relative to the expected haplotype. These rare variants generally differ by the addition or loss of a single repeat unit. (TIF) [file ppat.1003071.s001.tif]

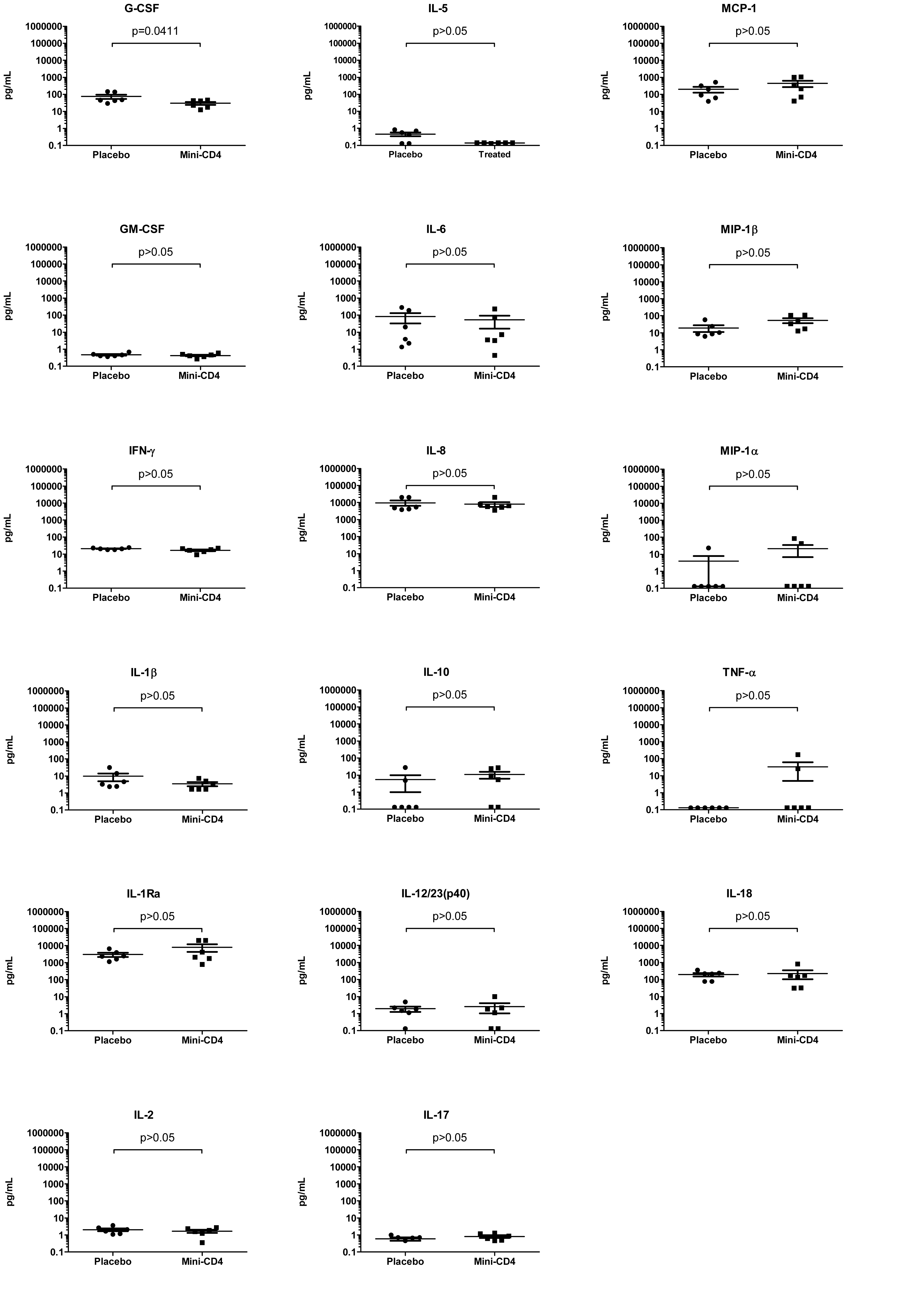

Supplement: Figure S2 — Cytokine and chemokine in macaque vaginal fluids after placebo- or M48U1 gel intravaginal administration. Vaginal fluids were collected 25 h after intravaginal application of either placebo- or M48U1-gel, i.e. 24 h after SHIV162P3 challenge. Individual data and mean with standard error of the mean (SEM) are presented. Group comparison was performed using non parametric Mann-Whitney test. Elevated TNF-α concentrations (not significant) were observed in two M48U1-treated animals (#16866: 26 pg/mL and #19831: 174 pg/mL). Both animals were protected from SHIV acquisition, suggesting that such TNF-α levels were not associated with enhancement of infection. (TIF) [file ppat.1003071.s002.tif]
